# Clinical management and nursing care for patients with tracheostomy following traumatic brain injury

**DOI:** 10.3389/fneur.2024.1455926

**Published:** 2024-12-20

**Authors:** Xiongyan Mao, Yuchan Zhou, Qiye Chen, Yelei Zhang

**Affiliations:** Department of Neurosurgery, Xishan People’s Hospital of Wuxi City, Wuxi Branch of Zhongda Hospital Southeast University, Wuxi, China

**Keywords:** tracheostomy, traumatic brain injury, decannulation, multidisciplinary team, nursing

## Abstract

Tracheostomy is a routine surgical procedure in patients with severe traumatic brain injury, which requires mechanical ventilation to maintain gas exchange and avoid hypoxemia. Inadequate tracheostomy timing, nursing care, and decannulation would lead to a series of complications, such as aggravated pneumonia and prolonged intubation. The effects of early tracheostomy versus late tracheostomy have been explored. And early tracheostomy is more likely associated with shorter hospital stays and fewer complications. But the relevant reports are controversial. A safe and fast tracheostomy decannulation would facilitate the recovery. However, there was a broad variability in the indications and timing of tracheostomy and decannulation. High-quality evidence is subsequently lacking. We conducted this review to address gaps in knowledge regarding the management strategy and nursing protocol in patients with tracheostomy and decannulation following traumatic brain injury. A multidisciplinary tracheostomy team containing nursing care was also discussed to provide the best service to these patients.

## Introduction

Patients suffered from severe traumatic brain injury (sTBI, the list of abbreviations was shown in Table 1) usually require mechanical ventilation in the neurosurgical intensive care unit (ICU). Approximately 31.8% of patients in the TBI cohort have experienced tracheostomy, which is more frequent than patients in general ICU cohorts with rates of about 10% (1). As the state of unconsciousness is persistent, tracheostomy is commonly performed to maintain gas exchange to avoid hypoxemia and accelerate liberation from mechanical ventilation. With the stable airway access provided, the tracheostomy tube could also be convenient for suctioning (2). The main indications for tracheostomy in patients with sTBI include absence of protective airway reflexes, impairment of respiratory drive, prolonged mechanical ventilation, reduction of dead space to facilitate ventilatory weaning, and difficulties in managing secretions (1, 3). However, optimal timing for tracheostomy is still controversial.

**Table 1:** List of abbreviations.

ABI	Acute brain injury
CRS-R	Coma recovery scale-revised
ET	Early tracheostomy
GCS	Glasgow Coma Scale
ICU	Intensive care unit
LT	Late tracheostomy
LOS	Length of stay
mPATH	Multiprofessional acute trauma health care
sTBI	Severe traumatic brain injury

A safe and fast decannulation in tracheostomized patients with sTBI is considered as a main rehabilitative goal. However, there was substantial heterogeneity in tracheostomy care and decannulation practices among the different centers (4). In this study, we reviewed the effects of tracheostomy, tracheostomy timing, and decannulation timing to provide valuable information for clinicians. We further discussed the role of a multidisciplinary tracheostomy team, especially the nursing care protocol, in the clinical management of tracheostomized patients with sTBI.

## The effects of tracheostomy on patients with sTBI

It has been reported that tracheostomy has advantages in improving outcomes of patients with sTBI compared with prolonged endotracheal intubation. Villemure-Poliquin N et al. (5) conducted a retrospective multicenter cohort study and examined the potential benefits of tracheostomy versus prolonged endotracheal intubation. Tracheostomy was shown to be associated with decreased 30-day mortality. The increased survival owing to tracheostomy was also confirmed by Stephen S et al. (6). Specifically, tracheostomy could comfort the patients by reducing oropharyngeal irritation, reducing sedative administration, and achieving more autonomy earlier (7). Besides, tracheostomy could decrease the risk of ventilator-associated pneumonia and ventilator-induced lung injury (8). Patients with long-term mechanical ventilation could also get positive weaning and shorter duration by tracheostomy. As an invasive procedure, tracheostomy would inevitably have risks for the airway, including tracheomalacia, hemorrhage, and tracheal stenosis (9). However, tracheostomy would still be recommended for sTBI patients when weighing the benefits and risks (10).

## The timing of tracheostomy for patients with sTBI

Conventionally, tracheostomy is commonly used for patients with sTBI under the condition of Glasgow Coma Scale (GCS) score ≤ 8 and these who rely on a mechanical ventilator for more than 7 days (11, 12). According to the recommendations of the European Society of Intensive Care Medicine consensus (13), tracheostomy is strongly recommended in mechanically ventilated patients with acute brain injury (ABI) who have failed one or several trials of extubation. It should also be considered in mechanically ventilated patients with ABI who have persistently reduced consciousness levels (weak recommendation).

Although tracheostomy is generally indicated for patients with sTBI, the timing of tracheostomy for patients with sTBI is unclear (14). Tracheostomy is usually defined as early tracheostomy (ET, performed within 7 days of admission) or late tracheostomy (LT, performed more than 7 days after admission). A previous study classified ET and LT as tracheostomy performed ≤10 days and > 10 days after tracheal intubation, respectively (15). Another study defined ET and LT as tracheostomy performed ≤3 days and > 3 days after admission, respectively (16). The effects of early tracheostomy versus late tracheostomy have been explored. But the results are controversial. According to a retrospective, 15-year observational cohort study from January 1990 to December 2005, 3,277 patients with TBI were found to have a tracheostomy (17). The investigation indicated a relationship between tracheostomy timing and prognosis, and suggested that ET may lead to a better overall clinical outcome than LT. Similarly, Shibahashi K et al. (18) explored the performance of earlier tracheostomy (within 72 h of admission) and found a decrease in the duration of mechanical ventilation and length of stay (LOS) in patients with TBI, with acceptable mortality. Besides, a propensity-matched analysis on children’s patients with sTBI and a retrospective cohort study on adult patients both showed that ET was related with shorter hospital LOS and fewer complications (8, 19). Along with a decreased risk of pneumonia, a lower risk of deep venous thrombosis, and decubitus ulcer, a decreasing trend of pulmonary embolism was observed in ET. A lower incidence of gram-negative microorganism-related nosocomial pneumonia and shorter antibiotic duration use were also identified in ET (20). In a meta-analysis comparing ET and LT in sTBI or stroke, the mean time to tracheostomy in the ET cohort was 7.1 ± 0.00 days and 15.3 ± 0.01 days in the LT cohort. ET was shown to reduce the risk for ventilator-associated pneumonia and decrease mechanical ventilation duration in ICU and hospital LOS (21). Another meta-analysis comparing ET and LT in sTBI displayed similar results (10). However, a recent US national analysis showed that mortality was slightly higher in ET than in LT, while other benefits from ET notably existed (22). Villemure-Poliquin N et al. (5) revealed no effect on mortality was observed when comparing ET and LT. Relevant studies on time to tracheostomy in patients with severe TBI in Table 2.

**Table 2:** Studies on time to tracheostomy in patients with severe TBI.

Authors/published year/journal	Aims	Study design	*N*	Primary results
Amirhossein et al. (22)/2023/Neurocrit Care	To compare the effect of ET versus LT in patients with TBI.	A retrospective cohort of inpatient study.	2,397 patients with ET (<7 days from admission) and 4,041 with LT (≥7 days from admission).	The patients with ET had a shorter length of stay as compared LT (*p* < 0.001) and had a lower hospital charge (*p* < 0.001). The mortality was higher within the ET group compared with the LT group (*p* < 0.001). Patients in the LT had higher odds of developing any infection (*p* < 0.001), emerging sepsis (*p* < 0.001), pneumonia (*p* < 0.001), and respiratory failure (*p* = 0.004)
Villemure-Poliquin N et al. (5)/2023/Can J Anaesth	To compare the effect of tracheostomy and prolonged intubation on patients’ outcomes, and to evaluate the tracheostomy timing on outcomes.	A retrospective multicentre cohort study.	374 with a tracheostomy and 609 with intubated remained;144 with an ET and 233 with a LT.	Tracheostomy was associated with a reduction in 30-day mortality (HR, 0.33; 95% CI, 0.21 to 0.53) compared with prolonged intubation. No effect on mortality was observed when comparing ET vs. LT procedures.
CENTER-TBI ICU Participants and Investigators (1)/2020/Intensive Care Med	To assess the effect of tracheostomy and its timing on patients’ outcomes.	A prospective observational longitudinal cohort study from CENTER-TBI.	1,358 included TBI patients and 433 with a tracheostomy. 180 with ET (≤7 days) and 253 with LT (>7 days).	Patients with a LT were more likely to have a worse mortality and poor neurological sequels (*p* = 0.018), and LOS (38.5 vs. 49.4 days, *p* = 0.003).
Cory et al. (19)/2019/J Trauma Acute Care Surg	To determine if ET is associated with decreased length of stay and fewer complications in children with sTBI.	A retrospective propensity score matching study.	168 with ET and 190 with LT.	ET was associated with fewer ventilator days (RR [95% CI] 0.55 [0.46, 0.65]), and a shorter hospital length of stay (0.62 [0.53, 0.72]). ET was also associated with less frequent pneumonia (OR 0.44 [0.26, 0.76]), and venous thromboembolism (0.20 [0.07, 0.57]). No significant differences in mortality (1.26 [0.46, 3.49]) were observed
Lu W et al. (16)/2019/J Craniofac Surg	To find the optimal time for tracheostomy in sTBI.	A retrospective study.	51 with ET (<3d) and 47 with LT (>3d).	The NICU stay, hospitalization stay, and antibiotic use time of patients in the ET group were shorter than those in the LT group (*p* < 0.05). The pneumonia rates and the cost in the ET group were lower than those in the LT group (*p* < 0.05). The complications and mortality were not statistically significant (*p* > 0.05).
Shibahashi K et al. (18)/2017/Br J Neurosurg	To examine the effects of tracheostomy performed within 72 h after admission in TBI patients.	A retrospective cohort study.	40 were in the ET group (≤72 h) and 51 were in the control group (>72 h).	The duration of mechanical ventilation and LOS in ICU were significantly less in the ET group than in the control group. The 30-day mortality rates were 3 and 8% for the ET and control groups, respectively.
Khalili H et al. (11)/2017/World Neurosurg	To compare the effects of ET versus LT on TBI-related outcomes and prognosis	A retrospective study.	53 with ET (≤6 d) and 99 with LT (>7 d).	Patients with ET had a significantly lower hospital stay (46.4 vs. 38.6 days; *p* = 0.048) and intensive care unit stay (34.9 vs. 26.7 days; *p* = 0.003). Mortality rates were not significantly different between the 2 groups (*p* > 0.99).
Aziz S et al. (8)/2014/J Trauma Acute Care Surg	To define the optimal timing of tracheostomy in patients with sTBI.	A retrospective observational cohort study.	873 with ET (≤8 days) and 938 with LT (>8 days).	ET was associated with fewer mechanical ventilation days (RR, 0.70; 95% CI, 0.66, 0.75), shorter intensive care unit stay (RR, 0.70; 95% CI, 0.66, 0.75), shorter LOS (RR, 0.80; 95% CI, 0.74, 0.86), and lower odds of pneumonia (OR, 0.64; 95% CI, 0.51, 0.80), deep venous thrombosis (OR, 0.53; 95% CI, 0.37, 0.78), and decubitus ulcer (OR, 0.43; 95% CI, 0.26, 0.71). No significant difference in pulmonary embolism (OR, 0.52; 95% CI, 0.24, 1.10). Hospital mortality was similar between both groups (OR, 1.25; 95% CI, 0.80, 1.96).
Wang HK et al. (20)/2012/Injury	To examine the impact of the tracheostomy timing in patients with sTBI.	A retrospective analysis.	16 were in the ET group (≤10 d) and 50 were in the LT group (>10 d).	ET a shorter duration of ICU LOS (*p* < 0.001), lower incidence of gram-negative microorganism-related nosocomial pneumonia (*p* = 0.001), and shorter duration of antibiotic use (*p* < 0.001).
Rizk EB et al. (17)/2011/Neurocrit Care	To determine the impact of tracheostomy timing on outcomes in sTBI.	A retrospective observational cohort study.	1,577 with ET (<7d) and 1,527 with LT (>7 d).	ET was more likely to be functionally independent at discharge (*p* = 0.001) and have a shorter LOS (*p* < 0.0001). However, LT were approximately twice as likely to be discharged alive (*p* < 0.0001).
Ahmed N et al. (14)/2007/Surg Infect (Larchmt)	To determine the impact of ET and LT in patients with sTBI.	A retrospective cohort study.	27 with ET and 28 with LT.	ET group had a significantly shorter stay in the ICU than late group (19.0 +/− 7.7 vs. 25.8 +/− 11.8 days; *p* = 0.008). There was no difference between the groups in ventilator days (15.7 +/− 6.0 vs. 20.0 +/− 16.0 days; *p* = 0.57). There were no significant differences between the groups regarding overall mortality (15% vs. 4%; *p* = 0.19).

ET, early tracheostomy; LOS, longer length of stay; LT, late tracheostomy; sTBI, severe traumatic brain injury.

In general, priority seems to be given to ET for patients with sTBI rather than delayed tracheostomy or LT. However, high-quality evidence is lacking. In 2020, an international consensus panel attempted to provide a recommendation regarding the optimal timing of tracheostomy in patients with ABI. But they failed due to contradictory and low-quality evidence (13). Following that, results from CENTER-TBI as a prospective observational longitudinal cohort study on patients with TBI were reported (1). The study demonstrated that LT was more likely to have a worse neurological outcome, poor neurological sequels, and longer LOS. Due to various tracheostomy timing, different brain injury severity, and a mix of isolated brain injury versus multiple injury, there was a broad variability in current studies. Standardized comparisons are needed in future research. Given the variable indications for mechanical ventilation and the different underlying lung mechanics in patient subgroups, more detailed information is needed to implement guidelines (23).

## The timing of decannulation for patients with sTBI

The majority of patients with tracheostomy who survive to hospital discharge could be successfully decannulated (24). An early decannulation could avoid secondary complications, such as respiratory infections and airway obstructions, to improve clinical outcomes and facilitate the recovery (25). However, the indications and optimal time for decannulation remain unclear. According to a systematic scoping review on critically ill patients in mixed ICU, the assessment criterion differed as informed consent, clinical stability, airway patency, physiological decannulation, swallowing assessment, consciousness level, effectiveness of cough, and clearance of secretions (26). However, it would not be adequate for patients with sTBI. Because patients with sTBI are usually in a coma, and the ability of self-body control and executing simple voluntary tasks are lost.

Weaning protocols are difficult for decision-making. There are numerous and confounding factors to judge. For example, common comorbidities such as diabetes and acute kidney injury would influence the chances for decannulation in patients with sTBI (27). Despite that, identifying efficient factors for time to decannulation is essential for safe weaning. It is reported that the predictive factors for safe removal of the tracheal tube in patients with ABI including high neurological status, TBI rather than stroke or anoxic brain lesions, younger age, effective swallowing, an effective cough, and the absence of pulmonary infections (28). Independent breathing and airway protection may indicate successful decannulation in patients with sTBI. Perin C et al. (29) found that mean expiratory pressure, spontaneous cough, and cough strength were positive predictors of tracheostomy tube removal. However, in a decannulation study on 74 patients with ABI, airway patency, cough reflex test, SpO2, and GCS ≥ 8 showed high specificity but low sensitivity (30). Shrestha KK et al. also proposed that consciousness level based on GCS score was not significant in successful decannulation (31). Meanwhile, a higher coma recovery scale-revised (CRS-R) score was suggested to accurately evaluate the state of consciousness, which was demonstrated to be associated with a higher probability of decannulation (32). Thus, accurate parameters for accessing consciousness need to be used for studies on decannulation indications in depth.

Several methods have been observed to contribute to successful decannulation. Lanini B et al. (33) emphasized the roles of flexible bronchoscopy and thought it would guide successful tracheostomy weaning. Zanata Ide L et al. (34) thought that phonation and coughing were helpful for early tracheal decannulation in 20 patients with TBI. Moreover, the swallowing ability was verified in a retrospective study with a Danish population of 324 participants. Eskildsen SJ et al. (35) found that an improvement in swallowing ability during the initial 4 weeks of rehabilitation was associated with an 8.2-day reduction in time to decannulation. This study concluded that swallowing ability is a potentially significant factor with reduced time to decannulation. Relevant studies on time to decannulation in patients with severe TBI in Table 3. In general, the clinical criteria for tracheostomy decannulation in patients with sTBI included: (1) toleration of tracheostomy tube capping for 72 h (36); (2) Absence of severe dysphagia, defined as the ability to manage secretions (37); (3) endoscopic patency of airways (lumen diameter > 50%) (38); (4) swallowing instrumental assessment (penetration assessment scale 5, no aspiration events); and (5) blue dye test (absence of blue traces) (30).

**Table 3:** Studies on time to decannulation in patients with severe TBI.

Authors/published year/journal	Aims	Study design	*N*	Evaluation for decannulation	Outcomes
Eskildsen SJ et al. (35)/2024/Respir Care	To identify significant factors for time to decannulation in subjects with tracheostomy after TBI.	A retrospective register-based cohort study.	324 patients	Associations between selected explanatory variables representing demographic and clinical characteristics and time to decannulation were analyzed using linear regression models.	An improvement in swallowing ability during the initial 4 weeks of rehabilitation was associated with an 8.2 d reduction in time to decannulation (95% CI −12.3 to −4.2, *p* < 0.001).
Jenkins R et al. (27)/2020/Brain Inj	To assess variables associated with decannulation in patients with TBI.	A retrospective study.	79 patients	Patients decannulated prior to 90 days were compared with patients who remained cannulated. Variables prior to tracheostomy and throughout hospitalization were used.	Variables prior to tracheostomy associated with decannulation included diabetes (HR, 0.15; 95% CI, 0.03–0.84; *p* = 0.03), craniotomy (HR, 0.25; 95% CI, 0.06–1.02; *p* = 0.05) and acute kidney injury (AKI) (HR, 0.06; 95% CI, 0.01–0.48; *p* = 0.01).
Hakiki B et al. (32)/2020/Arch Phys Med Rehabil	To identify the effect of some clinical characteristics on decannulation success in severe ABI.	A retrospective study.	351 patients with severe ABI.	Variables collected at admission during clinical examination, conducted by trained and experienced examiners were collected for analysis.	Absence of pulmonary infections (*p* < 0.001), sepsis (*p* = 0.001), tracheal alteration at the fibrobronchoscopy examination (*p* = 0.004) and a better state of consciousness at admission (*p* = 0.001) were associated with a higher probability of decannulation.
Enrichi C et al. (30)/2017/Respir Care	To find the most sensitive and specific clinical criteria for decannulation in ABI.	A cross-sectional experimental study.	74 patients	Control group: tracheostomy tube (TT) capping 72 h, no severe dysphagia, ability to manage secretions. Experimental group: TT capping ≥72 h, endoscopic patency of airways (lumen diameter > 50%), swallowing instrumental assessment, blue dye test, voluntary cough (PCF > 160 L/min), cough reflex, number of tracheal suctions, oxygen saturation > 95% ambient air, LOC (insufficient when the GCS was consistently ≤8)	Parameters showing the highest values of sensitivity and specificity, respectively, were tracheostomy tube capping (80, 100%), endoscopy assessment of airway patency (100, 30%), swallowing instrumental assessment (85, 96%), and the blue dye test (65, 85%). All these were combined in a clinical cluster parameter, which had higher sensitivity (100%) and specificity (82%).
Perin C et al. (29)/2017/Int Arch Otorhinolaryngol	To identify the factors associated with the outcome of tube removal in severe ABI patients.	A retrospective study	45 patients	Variables including demographic characteristics, GCS, cause of hospitalization, duration of Neurorehabilitation Ward hospitalization, comorbidities, kind of tracheostomy tube used, SpO2, pulmonary secretion, respiratory pressure, respiratory frequency and presence of spontaneous cough, cough strength, and blood gas analysis.	Mean expiratory pressure, presence of spontaneous cough, and cough strength. Provoked cough and GCS were not predictive of weaning success.
Zanata et al. (34)/2014/Int Arch Otorhinolaryngol	To accesses the applicability of a protocol for tracheal decannulation	A prospective observational study	20 patients	Informed consent, adequate LOC ≥ 8, phonation (orally responsive or nonresponsive), swallowing, secretions (amount, aspect and color), coughing, toleration of capping/cuff deflation	The protocol was relevant to establish the beginning of the decannulation process.
Matesz et al. (38)/2014/Orv Hetil	To describe the safe tracheostomy decannulation method of patients with brain injury during rehabilitation.	A prospective, descriptive study	20 patients	Consent, clinical stability (BP, pulse and peripheral oxygen saturation), bronchoscopy-aerogastric reflexes, state of vocal cords and possible stenosis were observed before decannulation, swallowing and speech assessment.	During the procedure successful decannulation was performed in 13 patients. Decannulation occurred 62 days after tracheostomy on average. The involvement bronchoscopy was feasible.
Shrestha et al. (31)/2012/Nepal Med Coll J	To evaluate gradual vs. abrupt techniques for successful decannulation in tracheostomised patients with sTBI.	A prospective case–Control study.	118 patients	Time since tracheostomy, timing of decannulation, Glasgow Coma Scale, amount of secretions, breath holding time, CXR and STN radiographs and cough reflex were all assessed.	Sixty-eight patients were decannulated gradually and 50 abruptly. Of the various factors assessed, only cough reflex, number of suctioning required per day, radiograph STN and use of antibiotics for more than 7 days were found to be statistically significant.

ABI, acquired brain injury; ET, early tracheostomy; LT, late tracheostomy; LOC, level of consciousness; sTBI, severe traumatic brain injury.

## Multidisciplinary tracheostomy team

Inadequate tracheostomy timing, nursing care, and unsafe decannulation would lead to complications, such as aggravated pneumonia, prolonged intubation, or induced paroxysmal sympathetic hyperactivity (39). Therefore, a multidisciplinary team trained and qualified is essential. Patients would benefit from a specialized multidisciplinary tracheostomy team. LeBlanc J et al. (40) retrospectively compared the effect of a multidisciplinary tracheostomy team from 27 patients before implementation of the tracheostomy team approach and 34 patients followed by the team. The team comprised trauma surgeons and residents, respiratory therapists, speech-language pathologists, and clinical nurse specialists. The results suggested that patients in the multidisciplinary group had a significantly shorter LOS and decreased time to decannulation. Based on research in the Level I trauma center of Carolinas Medical Center of America, Alvin et al. (41) investigated whether the multidisciplinary team would decrease LOS. The team was called a dedicated multiprofessional acute trauma health care (mPATH) team, consisting of a physical, occupational, speech, and respiratory therapist, nurse navigator, social worker, advanced care provider, and physician. They retrospectively compared the patients in the year before the mPATH team was established (*n* = 60) to those in the first full year following the team implementation (*n* = 70). As the primary endpoint, LOS obviously decreased due to the successful team. Meanwhile, a cost savings of US $11,238 per index hospitalization was observed. Thirty-day readmission and mortality rates had no significant change. Hence, the involvement of a multidisciplinary team is feasible during the entire hospitalization from tracheostomy to decannulation. However, it should be noted that the population sample size of the above study is limited, and more prospective research evidence is needed.

## Nursing care

In a recent methodological study conducted in a public hospital in the city of Belém in Brazil (42), 34 nursing professionals participated in this survey. Developed through two phases: (I) target audience characterization and (II) technology development, and guided by the 5W2H management tool: 1 – What; 2 – Who; 3 – When; 4 – Where; 5 – Why; 1 – How; 2 – How Much, investigators thought the educational demands and continuing health education, with an emphasis on standardizing care through a protocol, were important for critical patients with tracheostomy. Although the study was limited by the lack of content validity by expert judges, appearance validity by design judges, semantic validity by the target audience, and usability assessment, clinical significance may truly exist. Because they appealed that care protocols and educational technologies were effective tools to address inconsistent topics in assisting patients with tracheostomy in NICU. A cross-sectional descriptive study involving a self-administered questionnaire conducted in a tertiary medical center in Saudi Arabia also recommended continuous training and competency evaluation for delivering optimal nursing care (43).

Tracheostomy is an invasive procedure and requires the participation of specialized multidisciplinary tracheostomy team collaboration (44). Among those, if the nursing work is not in place, various complications could arise, and the clinical prognosis would be affected. Hence, evidence-based nursing for patients with tracheostomy is beneficial to form standardized practices and prevent complications (45).

According to our experience, an individualized and feasible respiratory care plan should be developed by the supervising nurse based on the patient’s condition. Firstly, different body postures should be used for patients with different disease conditions. For example, patients with irritating cough should be given a semi-recumbent position. Secondly, the suction method should be adjusted based on coughing and changes in airway pressure. The appropriate suction tube should be selected, and airway patency should be ensured. Each suction time should be approximately 15 s to prevent situations such as hypoxia and suffocation caused by prolonged suction. Thirdly, attention should be paid to airway humidification. After tracheostomy, the patient’s respiratory tract is in direct contact with air. Therefore, sodium chloride solution should be used for humidification. Simultaneously, a tracheostomy mask could be used to achieve a humidification effect. The gas could be humidified and filtered to protect the tracheal mucosa. Finally, complication care for stoma is important. Nurses should strengthen nursing patrols, check the incision, and prevent infection. The incision needs to be kept clean, the tracheal cuff should be replaced promptly, and disinfected with alcohol or iodine solution. Observe the incision for secretions, collect and culture bacteria, and use sensitive antibiotics to prevent infection.

## Discussion

Tracheostomy and its decannulation are common procedures for patients with sTBI. Although ET is mostly reported to be associated with decreased LOS and fewer complications, mortality remains controversial. The different outcomes reported in different studies are likely associated with the various patient models used. Evaluations for ET or LT are more effective when considering 7 days as a cut-off point. Further investigation into standardized patient models could provide more precise insights into the optimal timing for tracheostomy and improve patient outcomes across diverse clinical settings. In addition, the current evidence on protocols for the assessment of tracheostomy decannulation is inadequate. Most recommendations are insufficient due to time bias, population bias, or the small sample size owing to the nature of the study. With the results from CENTER-TBI proving that ET can improve patient outcomes, more prospective randomized controlled trials exploring the timing of tracheostomy and decannulation are warranted to provide clinical guidelines.

The multidisciplinary tracheostomy care is important and its scope is expanding in order to manage the complex needs and expectations of tracheostomy patients. Initiatives such as the Global Tracheostomy (Quality Improvement) Collaborative^1^ have the potential to collect meaningful patient-level data around the quality of care delivered (46). Quality improvement programs such as this can deliver data that are relevant to patients and their families, multidisciplinary health care professionals and also hospital administrators that can comprehensively benchmark the effectiveness of multidisciplinary tracheostomy care in the future (47). Even when overlapping with other roles from the multidisciplinary team, specialist nursing programs including continuous training and competency evaluation have been shown to be a cost-effective method of improving hospital-wide tracheostomy care.

There are some limitations in this review. Firstly, the included literatures were relatively insufficient. Several researches published before 2000 years were ruled out. And only English literature has been adopted. Articles in other languages such as Chinese, German, and French were not available. Secondly, we only focused on the results and conclusions of the existing research, and did not further analyze the data of the study comprehensively. In addition, the evidence of the multidisciplinary tracheostomy team and nursing care were not powerful. Therefore, some insights may be controversial.

## Conclusion

An ET performed within 7 days of admission in TBI patients could increase the opportunity of patient’s early rehabilitation. The toleration of tracheostomy tube capping, the ability to manage secretions, the endoscopic patency of airways, the swallowing ability, and the blue dye test may be potentially factors for early decannulation decision-making. A multidisciplinary tracheostomy team with specialist nursing care is crucial to improve clinical outcomes and prevent complications.

## Footnotes

^1^ www.globaltrach.org
